# The New York Homœopathic Union

**Published:** 1897-11

**Authors:** 


### THE NEW YORK HOMŒOPATHIC UNION.

The regular monthly meeting of the New York Homœopathic Union was held at 62 West Forty-ninth Street, New York, Thursday, October 21st, 1897, the President, Edmund Carleton, M. D., in the chair. This was the first meeting of the season of 1897-98.

Members present: Baylies, Carleton, O'Brien, Powel, Young, Wilcox; also medical students Spencer Carleton and Mayer. Regrets were received from Drs. Leverson and Talcott.

The recently adopted plan of devoting one-half of the meeting to philosophical, and the other half to clinical topics was continued in force, the clinical part being considered first on this occasion. Consideration of calculus was continued from the last meeting.*

Dr. Carleton reported a recent case relieved promptly by Belladonna. Pains were in sharply defined paroxysms, coming and going quickly, and excruciatingly severe while they lasted. The following day a stone was passed, about the size of a large pea, and shaped approximately like a kidney. It weighed millegr. xxxvii. Mr. Spencer Carleton analyzed it,

---

*See The Homœopathic Physician for July, 1897, pages 274 to 277.

and found it composed mostly of uric acid, with a little calcium urate.

Dr. Baylies spoke of a case relieved with Colocynth. The characteristic colic causing the patient to double over forward was present. A large, red stone, of uric acid formation, was expelled, and there has been no trouble since.

Dr. O'Brien—I should like to ask the President if he objects to the application of heat in such cases.

Dr. Carleton—There can be no objection to hot blankets, hot water-bags, or any other method of applying dry heat when desired, as it commonly is, by the patient. Circulation of blood, relaxation of spasm, and general comfort are thereby promoted. These applications are not medicinal, and not curative. Only the homœopathic medicine cures. The objection to moist heat is that once begun it must be continued at frequent intervals, and later the patient is liable to take cold.

Dr. Baylies had a case of chronic diarrhœa that had a curious circumstance attending it. The mother took on the daughter's condition sympathetically, and as she improved the mother improved also.

Dr. Carleton was summoned in haste to a middle-aged woman of sedentary habits. Pain extreme and continuous along left ureter, gradually moving toward bladder. Irritable mentally, with red face and cold feet; wanted to be covered warmly, and also have artificial heat; chilly on motion. Nausea, but no thirst. She sent for me because she had heard that Homœopathy could relieve without opiates, which the old school could not do. She received *Nux-vom.* 200th in water every five minutes. In half an hour relief came. Then medicine was given every two hours until well, and then stopped. Next day gravel passed.

Dr. Young—Which causes more trouble, kidney or liver calculus? My experience has been that liver is more painful. It will cause tonic spasms.

Dr. Powel—If that is so I want none. Kidney stone is bad enough. I had an attack with this symptom; could not bear anything on abdomen. *Lach.* 1400 relieved in five minutes. Another time, desire for warmth, but restless tossing about; constant moistening of lips with water relieved.

Dr. Baylies—Have you had any experience with olive oil? Before I became a homœopathist I used large doses of it, followed by castor oil, which gave some relief, but caused green passages.

Dr. Young—Is not relief due then to relaxation, for how could the oil reach the duct?

Dr. Powel—Dr. Swan says that olive oil contains cholesterin, which is absorbed. The oil itself is but a lubricant. I have used the oil after the attack is over.

Dr. Young—What do you think of the last act of the Board of Health declaring that leprosy is not contagious?

Dr. Carleton—That is the modern variety. The old, white, Bible variety we do not see here now.

Dr. Spencer Carleton—I saw the cases that Dr. Young alludes to, and the skin was yellow and pigmented.

Dr. Young—I had a cancer case which took a leprous form. It affected one leg and spread to the thigh, then over abdomen, gradually changing from first red, thickened and burning skin, to whitish, hard and leathery. Scales formed, showing pustules underneath. It continued spreading until fever resulted in death. History of family showed consumption, scrofula, and cancer. I tried remedies, but they relieved only for a time.

Dr. Carleton—If I had a case of the real, old-fashioned white leprosy, such as is mentioned in the Bible, I should think of Carbolic-acid. Its pathogenesis shows both whiteness and anæsthesia.

Dr. Baylies—I should like to ask Dr. Young if his case was like ichthyosis?

Dr. Young—Not wholly. Sometimes scabs would fall off, leaving a lardaceous base.

The subject of intermittent fever was then considered.

Dr. Carleton—A middle-aged lady came to this city recently from her summer residence in a very healthy town, or rather township. All conditions there were sanitary. The family began to reside in one of our best hotels. The premises were in excellent condition, as proved by painstaking examination. However, the house fronted upon a thoroughfare which had been extensively ripped open of late, but had been nearly restored to its proper state before the arrival of the family. Five days later the lady became ill. Presumably the city is at fault. She had thirst and pain in the bones all night. The next morning she had a severe chill; then vomiting of a bitter substance; then fever, and lastly sweat. A dose of *Eupatorium-perfoliatum*, CM. [F.], finished the case at once. The next day there was a smart diarrhœa, and that was all.

This history has been given to you because the indications for the remedy were so clear. The next case has been selected, from an unusually large number observed this autumn, on account of the difficulty experienced in obtaining an accurate picture of the malady.

The subject was a middle-aged man who had contracted ague in the southwest some years ago, and had been dosed with Quinine and other drugs. He had never felt well since. The fever kept recurring every spring and autumn, and as often was suppressed, modified, and masked by improper medication. Partly in consequence of this he had formed intemperate habits. His business—that of a master mechanic—was seriously interrupted. When he came to my notice the symptoms were irregular and jumbled to a great degree. Failing to obtain a statement from the patient and his family after careful questioning, sufficiently full and definite to meet the requirements of a homœopathic prescription, I determined to watch an attack myself. The attacks were quotidian, tertian, seldom quartan, without any relationship to the time of day.

August 16th—Prodrome; aching bones, beginning early in the morning. Chill ("dumb") beginning in tips of fingers, 9. 30 A. M., lasted until noon, with thirst relieved by drinking. Dizzy and faint when moving, especially when sitting up. Wanted to be let alone and not answer questions. No appreciable fever. No sweat. During apyrexia very irritable, cross, restless, and thirsty.

The next paroxysm was two days later, beginning at 10 A. M. This time fever, with delirium, frontal headache, soreness of eyes extending back to occiput, and aching all over, especially the joints of fingers, wrists, and ankles. Felt all over as if pounded. Sweat brought partial relief. Felt badly the following day—stretching, yawning, tired, restless, going from one bed to another, then to the floor, a chair, and back to bed; hungry, but could not find the right thing to eat.

Three (?) days later, at 2 P. M., came a paroxysm all unannounced, somewhat resembling the first one, with sweat and imperfect apyrexia. It was evident to me, that nature was endeavoring to establish an untrammeled type. So the patient was amused with white powders, while we waited. He was extremely inapt and reticent when relating symptoms; and his family rendered but poor assistance. No good opportunity to "take the case" was had again until September 3d; and, meantime, the most confusing accounts of it were received. Then I saw the following:

10.30 A. M.—Thirst; drinking cold water caused chilly feeling all over. Soles felt bruised. 11 A. M.—Cold tips of fingers, which were blue; cold toes; then cold neck, chest, and arms. Aching wrists and ankles. Thirstless. Pulse, 94. Temperature, 100.5. 11.50 A. M.—Yawning and stretching; sleepy; eyes watery; felt better when sitting. When lying head became "crazy." That was his word, and I could not get any other. Wanted to lie down, but dared not for fear of the "crazy" sensation. Forehead and vertex hot all the time. 12 noon.—Forehead began to ache, with pain in right eye, which felt large. Sensation of cold gone, except in hands. Examination revealed less cold hands and other parts. 12.15 P. M.—Chill waning and fever coming on. Feet ached worse and felt hot. Chest felt contracted. Yawning and stretching continually. No thirst. 1 P. M.—Attack ended. No sweat. Apyrexia: Easily irritated by noise of children and trifling affairs. Loss of memory. "Surging, like a big buzz-saw," in left side of head. Thirst for cold water; drinking caused pain in and around heart. Two or three hours after paroxysm sharp, stitching pains in region of spleen, lasting about three hours. Slept well, but felt weary in the morning. Liver and spleen were found to be enlarged.

I made comparisons of all the remedies that have occurred to you while listening to these details, but could not find the simillimum; and was averse to zigzagging. The thirst, time of attack, frequency, modalities, and combinations, and especially the symptoms of the apyrexia—so important—did not present a characteristic and uncontradicted picture of any remedy. Then for a number of days the patient was out of my sight, reporting very imperfectly the changes in time and varying symptoms as they occurred.

September 19th I received this scrap of news: "Chill began about 2 P. M. and lasted until 4. No fever to speak of, but sweat on head and hands. Headache and nausea after chill." This helped somewhat, as to time of attack, its stages and modalities. I then sought the patient to get more information, being grimly determined to effect a cure if possible, but had no success, and was on the point of leaving him in disgust when the following was discovered: He had been drowsy before the attack; laid down and went to sleep, and awoke cold and shaking, the attack being well on. These facts, added to what had been obtained, made a picture of disease that was symmetrical, sufficiently full, and uncontradictory. The required "case" had been taken at last. A dose of *Lachesis* CM. [F.] cured promptly. He did not have another chill, and has been well since. He, his family, and friends unite in testifying that his health has been better since than for a number of years before.

The members then gave consideration to homœopathic philosophy. The following paper, by Dr. W. S. Talcott, was read:

### DISEASE AND DRUG ACTION.

W. S. Talcott, M. D.

